# Diabetes Mellitus and Stroke: Pathophysiological Connections and Therapeutic Potential of GLP-1 and GLP-1/GIP Receptor Agonists

**DOI:** 10.3390/pharmaceutics18050620

**Published:** 2026-05-19

**Authors:** Maria-Alexandra Paceana, Liliana Mititelu Tartau, Ianis Kevyn Stefan Boboc, Carmen Nicoleta Oancea, Anca Berbecaru-Iovan, Cezar Ilie Foia, Cosmin Gabriel Tartau, Maria Bogdan

**Affiliations:** 1 Doctoral School, University of Medicine and Pharmacy, 200349 Craiova, Romania; mariaalexandrapaceana@gmail.com; 2 Grigore T. Popa University of Medicine and Pharmacy Iasi, 700115 Iasi, Romania; cezar030699@gmail.com (C.I.F.); cosmin.tartau@gmail.com (C.G.T.); 3 Department of Pharmacology, Faculty of Pharmacy, University of Medicine and Pharmacy, 200349 Craiova, Romania; kevynboboc@gmail.com (I.K.S.B.); ancaberbecaru@yahoo.com (A.B.-I.); bogdanfmaria81@yahoo.com (M.B.); 4 Department of Biochemistry, Faculty of Medicine, University of Medicine and Pharmacy, 200349 Craiova, Romania; carmen.oancea@umfcv.ro

**Keywords:** diabetes mellitus, stroke, antidiabetic drugs, GLP-1 receptor agonists, GLP-1/GIP receptor agonists

## Abstract

Both diabetes mellitus (DM) and stroke are major global health challenges with high morbidity and mortality. DM is a major risk factor for stroke, contributing to both increased incidence and worse clinical outcomes. Incretin-based therapies, including glucagon-like peptide-1 receptor agonists (GLP-1 RAs), as well as dual agonists like tirzepatide, have demonstrated significant cardiovascular benefits, raising interest in their potential cerebrovascular effects. This narrative review examines the pathophysiological links between DM and stroke and summarizes recent clinical evidence on the efficacy of GLP-1 RAs and dual GLP-1/GIP receptor agonists (GLP-1/GIP RAs) in stroke prevention and management. Current evidence from large cardiovascular outcome trials supports the role of GLP-1 RAs in reducing major adverse cardiovascular events, including stroke, primarily in the context of primary and secondary prevention. Findings suggest that semaglutide and liraglutide may reduce non-fatal stroke incidence, decrease hospitalizations, and improve neurological outcomes in patients with prior stroke. Comparative analyses of major trials suggest that, although stroke reduction may be a class effect of GLP-1 RAs, meaningful differences exist between individual agents, likely due to variations in pharmacokinetics, receptor affinity, and study populations. Additionally, much of the evidence in acute stroke derives from early-phase or ongoing trials, warranting cautious interpretation. Novel therapies, including orforglipron and retatrutide, as well as combinations like Maridebart cafraglutide and CagriSema, may expand future therapeutic options for individuals at high cerebrovascular risk. GLP-1-based therapies show promising neurovascular effects, but large-scale, long-term studies are needed to define their role in post-stroke management and cerebrovascular risk reduction. Overall, GLP-1 RAs should currently be regarded primarily as agents for long-term vascular risk reduction rather than established therapies for acute stroke. While potential neuroprotective effects are emerging, these require confirmation in adequately powered randomized trials. Future studies should aim to identify the patient subgroups most likely to benefit and to determine whether specific agents confer advantages in acute cerebrovascular contexts. A better understanding of the mechanisms underlying potential neuroprotection will be essential to determine whether these therapies can be effectively integrated into stroke management strategies.

## 1. Introduction

The 11th edition of the IDF Diabetes Atlas (2025) identifies diabetes mellitus (DM) as one of the most rapidly escalating global health challenges of the 21st century, with projections estimating that the number of affected individuals will reach 853 million by 2050. Type 2 diabetes mellitus (T2DM) represents the predominant form, accounting for more than 90% of all diabetes cases worldwide. Currently ranked as the 8th leading contributor to global disease burden, T2DM is anticipated to rise to the second leading cause by 2050 [1]. Importantly, individuals with T2DM exhibit a substantially increased risk of cardiovascular complications, including a 52% higher risk of stroke; therefore, optimizing the management of this DM complication should remain a key priority for healthcare systems [1].

The purpose of this narrative review was to summarize current evidence regarding the pathophysiological links between DM and stroke, as well as the potential beneficial effects of glucagon-like peptide-1 receptor agonists (GLP-1 RAs) and dual GLP-1/GIP receptor agonists (GLP-1/GIP RAs) in stroke prevention and management. The synthesis aimed to integrate clinical and mechanistic evidence to provide a comprehensive overview of the topic and to identify current gaps in knowledge.

A comprehensive literature search was performed using the following electronic databases: PubMed/MEDLINE and Web of Science. The search was conducted for articles published in English between January 2020 and March 2026 to ensure inclusion of the most recent evidence. Eligible studies included randomized controlled trials, observational studies, meta-analyses, and systematic and narrative reviews.

The search strategy combined Medical Subject Headings (MeSH) terms and free-text keywords, including but not limited to: “diabetes mellitus”, “type 2 diabetes mellitus”, “pharmacotherapy”, “stroke”, “ischemic stroke”, “risk factors”, “drug repurposing”, “cardiovascular disease” “GLP-1 receptor agonists”, “GLP-1 RA”, “dual incretin agonists”, “GIP”, “tirzepatide”, “semaglutide”, and “liraglutide”.

Initially, titles and abstracts identified through the database search were screened to exclude clearly irrelevant articles. Subsequently, full-text versions of potentially eligible articles were assessed for inclusion. To ensure comprehensive coverage, the reference lists of all included articles were manually screened for additional relevant studies.

Given the narrative nature of this review, the selection of studies was not restricted by strict inclusion or exclusion criteria; the final selection of references was based on relevance, scientific rigor, and contribution to the overall understanding of the topic. No formal quantitative meta-analysis was conducted due to variability in study methodologies and endpoints.

Additional sources included ClinicalTrials.gov for ongoing and recently completed clinical trials, after which we searched PubMed for clinical trials’ published results using ClinicalTrials IDs. For already approved GLP-1 RA and GLP-1/GIP RA antidiabetic drugs, the search conducted on ClinicalTrials.gov used the following keywords: “semaglutide”, “liraglutide”, “dulaglutide”, “exenatide”, “tirzepatide” AND “stroke”. Studies conducted exclusively in healthy volunteers, as well as studies in which stroke was not included among the eligibility criteria, were excluded. The studies presented in Table 2 were considered eligible based on their direct relevance to stroke and the cerebrovascular profile of the investigated populations. Another search was performed on ClinicalTrials.gov using the following keywords: “CagriSema”, “maridebart cafraglutide”, “orforglipron”, “retatrutide” AND “stroke”, “atherosclerosis”, and “hypertension”. The potential therapeutic agents were selected according to their current relevance in the development of next-generation incretin-based therapies, prioritizing molecules undergoing active clinical investigation and demonstrating potential cardiovascular and/or cerebrovascular implications relevant to the objectives of the manuscript. The inclusion of the studies in Table 3 was based on their clinical relevance to cerebrovascular disease, with priority given to studies directly investigating stroke, populations at increased cerebrovascular risk, or atherosclerotic conditions associated with significant vascular risk.

## 2. Diabetes Mellitus

DM is a long-term condition affecting over 588 million people worldwide [1] primarily characterized by the body’s inability to maintain normal blood glucose levels. This occurs either because of pancreatic dysfunction affecting insulin synthesis or secretion, or due to an inadequate tissue response to insulin [2].

### 2.1. Types and Symptoms

**Figure 1:**
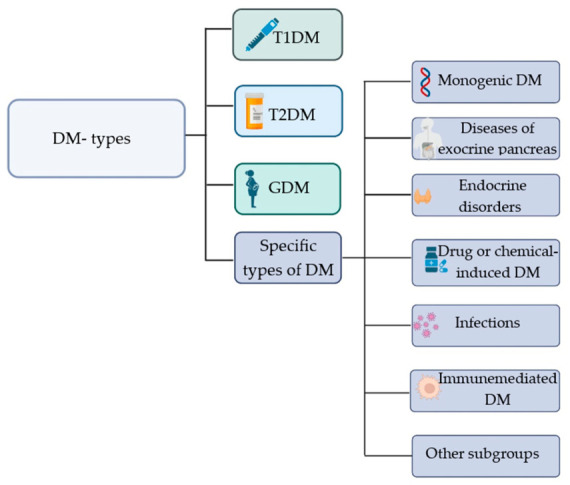
Classification of specific types of DM [3]. *Abbreviations*: DM—Diabetes Mellitus; T1DM—Type 1 Diabetes Mellitus; T2DM—Type 2 Diabetes Mellitus; GDM—Gestational Diabetes Mellitus. Created at https://BioRender.com.

The World Health Organization (WHO) has published an extensive classification of DM types, which includes type 1 diabetes mellitus (T1DM), T2DM, gestational diabetes mellitus, and other types of DM (often referred to as “specific” or “special types”) resulting from various causes (Figure 1) [3].

DM has several well-known symptoms, with little to no variation between its types. The main clinical manifestations of DM are polyuria, polydipsia and polyphagia [4]. T1DM typically presents with the following symptoms: constant thirst; frequent urination; lack of energy and fatigue; constant hunger; unintentional sudden weight loss; blurred vision and/or diabetic ketoacidosis (especially in pediatric cases) [4]. T2DM presents with similar symptoms; however, it is important to highlight its insidious onset, which explains why many patients remain undiagnosed or are diagnosed only after complications arise [1]. In comparison with T1DM, people with T2DM rarely develop ketoacidosis or weight loss; nevertheless, a hyperosmolar coma may occur, especially in elderly patients [3].

### 2.2. Pharmacotherapy

The main cause of glycemic imbalance in individuals with T1DM is almost absent β-cell function; therefore, the administration of insulin is crucial. Patients rely on an insulin regimen carefully chosen by their clinician, taking into consideration various lifestyle factors [5].

**Figure 2:**
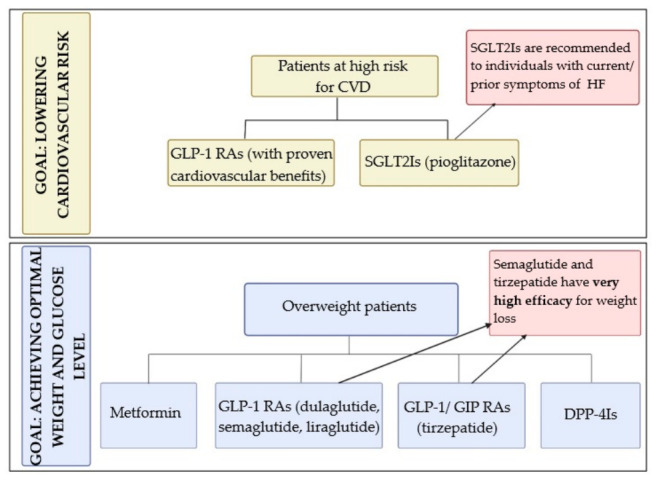
Pharmacological approach of T2DM [5]. *Abbreviations*: CVD—cardiovascular disease; GLP-1 RAs—glucagon-like peptide-1 receptor agonists; SGLT2Is—sodium–glucose-linked transporter inhibitors; HF—heart failure; GLP-1/GIP RAs—glucagon-like peptide-1 and glucose-dependent insulinotropic peptide dual receptor agonists; DPP-4Is—dipeptidyl peptidase-4 inhibitors. Created at https://BioRender.com.

The pharmacological management of T2DM should take into consideration comorbidities and frequently occurring complications, as presented in Figure 2. The two main comorbidities associated with T2DM are cardiovascular diseases (CVDs) and obesity, and addressing these comorbidities is an effective strategy for selecting the most appropriate therapeutic agents [5].

The pharmacological treatment of T2DM can include either insulin and non-insulin therapies or a combination of both. Non-insulin agents, oral or injectable, have become the standard of care for T2DM. Insulin therapy is usually initiated for patients with poor glycemic control despite dual or triple therapy with other antidiabetics [6].

Non-insulin agents are usually classified by taking into consideration their mechanisms and/or their chemical structure, as listed below.

#### 2.2.1. Insulin Secretagogues—Agents That Directly Stimulate the Secretion of Insulin in β-Cells

- Sulfonylureas (SUs) (glyburide, glipizide, and glimepiride)

SUs bind to sulfonylurea receptors (SURs) on pancreatic β-cells, triggering a cascade of events that ultimately lead to insulin secretion. SUs still represent a valid choice due to their safety profile and low cost, although studies have shown a higher incidence of hypoglycemia among patients using glimepiride compared to patients who were treated with other antidiabetics, for example, sodium–glucose-linked transporter-2 inhibitors (SGLT2-Is) such as licogliflozin or dapagliflozin [7].

- Glinides or meglitinides (nateglinide and repaglinide)

Glinides bind to a different site on SURs compared to SUs, but they determine the same cascade response leading to increased insulin secretion. An important aspect to highlight is that glinides exert their action depending on the level of glucose in the blood. Meglitinides are associated with a lower risk of hypoglycemia compared with SUs and they are short-acting [8].

#### 2.2.2. Agents That Improve Insulin Sensitivity

- Biguanides (metformin)

Metformin was widely used in Europe with regulatory approval from national authorities long before its later authorization by the European Medicines Agency (EMA) [9] and the U.S. Food and Drug Administration (FDA), which further consolidated its role in T2DM management. Currently, metformin is used by over 200 million patients with T2DM, being a first-line drug, especially for individuals who also suffer from obesity. Metformin indirectly stimulates adenosine monophosphate-activated protein kinase (AMPK), lowers cytosolic dihydroxyacetone phosphate and also raises cytosolic NADH/NAD ratio [10].

- Thiazolidinediones or glitazones (rosiglitazone and pioglitazone)

The mechanism of action of thiazolidinediones involves activation of peroxisome proliferator-activated receptors (PPARs). The agents included in this subgroup of antidiabetics are associated with a high risk of toxicity. Rosiglitazone is associated with an increased risk of cardiovascular events, leading to significant restrictions on its use [8]. Pioglitazone has been controversially linked to bladder cancer; however, available evidence has been insufficient to establish a definitive causal relationship [11].

#### 2.2.3. Agents That Decrease Intestinal Glucose Absorption

- α-Glucosidase inhibitors (acarbose and miglitol)

α-Glucosidase is an enzyme that contributes to the digestion of complex carbohydrates by inhibiting it, and acarbose and miglitol indirectly stop the process of transforming carbohydrates into simple monosaccharides [8]. This mechanism of action helps with the management of postprandial hyperglycemia [12].

#### 2.2.4. Agents That Increase Renal Glucose Secretion

- SGLT2Is (gliflozins)

SGLT2Is disrupt renal glucose reabsorption in the proximal tubules, thereby promoting the urinary excretion of significant amounts of glucose [13]. More recent studies showed that SGLT2Is also exert beneficial cardiovascular effects; a randomized controlled trial showed that dapagliflozin is able to reduce heart failure among patients with T2DM by approximately 27%, and the same beneficial cardiovascular effects were observed for canagliflozin and empagliflozin [14].

#### 2.2.5. Incretin-Mimicking Agents

- Dipeptidyl peptidase-4 inhibitors (DPP-4Is)

Currently, four DPP-4Is—sitagliptin, saxagliptin, linagliptin, and alogliptin—have obtained both EMA and FDA approval [8,15]. Their mechanism of action consists of inhibiting DPP-4 and, therefore, prolonging the action of incretin hormones. Studies describe DPP-Is as safe and effective therapeutical options for managing hyperglycemia in T2DM [16].

- Glucagon-like peptide-1 receptor agonists (GLP-1 RAs)

Glucagon-like peptide-1 (GLP-1) is an incretin hormone that exerts several important effects, including glucose-dependent stimulation of insulin secretion, induction of satiety, and delayed gastric emptying; GLP-1 RAs such as semaglutide, liraglutide, dulaglutide and exenatide mimic these effects and thereby help manage the metabolic imbalance in T2DM. The landmark trial STEP1 [17] demonstrated the substantial weight loss effect of semaglutide. Furthermore, studies have shown that GLP-1 RAs also confer cardiorenal benefits, which has encouraged their wider use, particularly given the large proportion of patients with DM who are at high cardiovascular risk [18].

- GLP1 and glucose-dependent insulinotropic peptide (GIP) dual receptor agonists (GLP-1/GIP RAs)

GLP-1 and GIP are two peptide hormones that function together to produce the incretin effect, which plays a crucial role in hormonal regulation [19]. Tirzepatide is a GLP-1/GIP RA that has been approved by the FDA and EMA [20] and is currently used for the treatment of both T2DM and chronic obesity. The SURMOUNT trials highlighted key findings regarding tirzepatide, demonstrating that once-weekly administration led to weight reductions ranging from 16% to 26.6% [21].

The pharmacological management of T2DM has evolved considerably, encompassing multiple drug classes with distinct mechanisms of action. Rather than functioning as isolated therapeutic options, these agents are increasingly selected based on their broader metabolic and cardiovascular effects, particularly in patients with comorbid conditions.

## 3. Stroke

Stroke is a clinical syndrome characterized by neurological deficits, which can be caused either by the occlusion of a blood vessel (ischemic type) or by the rupture of a vessel followed by hemorrhage (hemorrhagic type). It is categorized as a non-communicable disease (NCD) and was the third leading cause of death globally in 2021, according to WHO global statistics [22]. According to the 2025 Heart Disease and Stroke Statistics update published by the American Heart Association, the number of deaths caused by this disorder increased by 44% from 1990 to 2021, when a total of 7.25 million people died from stroke [23].

### 3.1. Types and Symptoms

Depending on the underlying cause, strokes can be classified into two types.

Ischemic stroke, the most prevalent, is usually caused by the occlusion of an artery, resulting in neurological deficits that may lead to permanent damage. Hemorrhagic stroke, on the other hand, is caused by the rupture of a blood vessel, leading to brain hemorrhage [24].

Symptoms of stroke may vary according to the type of stroke the patient has suffered. Although studies have identified possible patterns in clinical presentation that may help differentiate hemorrhagic stroke from ischemic stroke, neuroimaging remains the most accurate method for determining the type of stroke [25]. The most well-known methods for the early recognition of stroke are the BE-FAST and FAST methods. The acronyms stand for Balance, Eyes, Face, Arm, Speech, and Time. Studies have shown that both methods are useful but have low specificity [26].

### 3.2. Pharmacotherapy

Although acute ischemic stroke (AIS) requires immediate medical intervention, in most cases following the correct therapeutic approach takes time. Studies have shown that a 30-min delay in restoring adequate cerebral perfusion can decrease the likelihood of a good stroke prognosis by up to 10% [27]. The main aspects that should be taken into consideration are listed below.

*The time since the last known well (LKW)*

LKW represents the last time the patient was seen in a normal neurological state. If the patient experiences a stroke while sleeping, the LKW is considered to be the time they went to sleep or before. The usual therapeutic approach when the LKW is less than 4.5 h prior is to initiate intravenous (IV) thrombolysis, provided there are no contraindications. Alteplase and tenecteplase are usually considered first for IV thrombolysis [28].

*The severity of the neurological deficit*

A widely used assessment score for measuring the severity of neurological deficit post stroke is the National Institutes of Health Stroke Scale (NIHSS). Scores of 5 or below are generally associated with mild symptoms, and up to half of ischemic strokes cases present with such manifestations, leading to a therapeutic dilemma when determining the optimal treatment. A 2025 study concluded that treatment of ischemic stroke caused by large vessel occlusion (LVO) with an NIHSS score of 5 or lower should include either IV thrombolysis (if eligible) or antithrombotic therapy, rather than endovascular thrombectomy, which should be reserved for moderate to severe cases [29].

*Contraindications to IV thrombolysis*

Although IV thrombolysis is the first therapeutical option for post-stroke care, patients who are not eligible due to contraindications should start antiplatelet or anticoagulant treatment. Provided that there are no contraindications, studies have shown that the administration of aspirin within 48 h of stroke is beneficial for patients. When discussing anticoagulant therapy, a full dose of heparin administered for 10 days showed no additional therapeutic benefit compared to antiplatelet therapy [28].

*Results of neuroimaging*

Several neuroimaging techniques are used to identify specific pathological characteristics that can guide the most appropriate therapeutic approach. Non-Contrast Computed Tomography (NCCT) can demonstrate changes caused by neurological deficits in the middle cerebral artery territory using the Alberta Stroke Program Early CT Score (ASPECTS), a scoring system ranging from 0 to 10, where a score of 10 indicates little to no early ischemic changes. Other important neuroimaging modalities include contrast-enhanced CT angiography (CTA) and magnetic resonance angiography (MRA), showing crucial details such as the location of the occlusion [28].

### 3.3. Various Agents as New Therapeutical Options for Stroke

As individuals who have suffered a stroke are at high risk of permanent cognitive dysfunction [30], understanding the underlying pathophysiological mechanisms that occur post-stroke and applying targeted treatments accordingly is essential for limiting the pathological consequences of stroke.

Extensive studies have revealed a considerable number of therapeutic agents that can be repurposed for post-stroke recovery [31,32,33,34,35,36]. Identifying new therapeutic options for post-stroke recovery through drug repurposing offers the advantage of existing research available for most of the drugs discussed, thereby allowing new research efforts to focus on relevant pathophysiological targets while simultaneously reducing research costs [32].

Beyond repurposed drugs, several novel and investigational therapeutic agents have been explored for stroke’s treatment, targeting distinct pathophysiological mechanisms such as excitotoxicity, oxidative stress, endothelial dysfunction, neuroinflammation, and impaired cerebral perfusion. These agents [e.g., human urinary kallidinogenase, edaravone, glyceryl trinitrate, nerinetide, sovateltide, uric acid, and nelonemdaz] represent diverse pharmacological strategies aimed at improving neuroprotection and functional recovery [31,37,38,39,40,41,42,43]. Although these therapies differ in their molecular targets and clinical development stages, they collectively reflect a shift toward mechanism-driven interventions designed to complement reperfusion therapies [37].

While these diagnostic and therapeutic considerations are essential in acute stroke management, they do not fully address the underlying metabolic factors that influence stroke risk and recovery. In this context, the interaction between diabetes and cerebrovascular disease becomes particularly relevant, as it may open new avenues for targeted therapeutic interventions.

## 4. DM and Stroke—Connections

As medicine has developed, it has been found that DM and stroke are connected by a few pathways, indicating that individuals with DM have a significantly higher risk of stroke compared to individuals with a normal response to glucose [44]. Furthermore, research shows that diabetic patients may encounter more difficulties in the recovery period after stroke then individuals without DM [45].

### 4.1. Risk Factors

**Figure 3:**
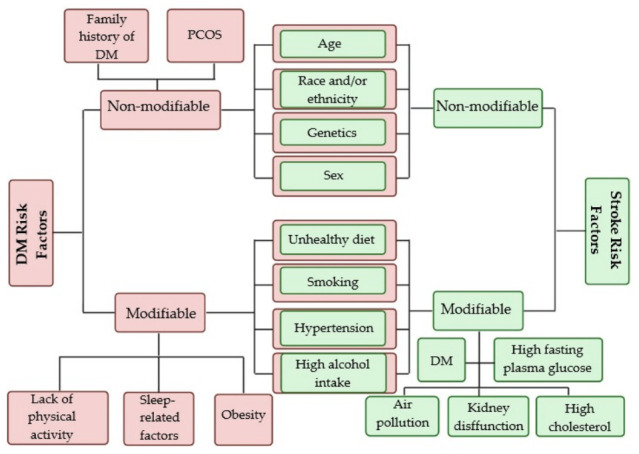
Risk factors of DM and stroke [5,46,47,48,49]. *Abbreviations*: PCOS—Polycystic Ovary Syndrome; DM—Diabetes Mellitus. Created at https://BioRender.com.

DM and stroke are complex disorders characterized by heterogeneous pathophysiological mechanisms, clinical subtypes, symptomatologies, and risk profiles. The risk factors associated with DM vary according to disease subtype, and their relative contributions and underlying mechanisms are not yet fully elucidated. Similarly, stroke remains a multifactorial condition with incompletely understood pathophysiological pathways; nevertheless, extensive research has identified a set of well-established risk factors. The main risk factors for both DM and stroke, which are commonly classified into modifiable and non-modifiable categories, are summarized in Figure 3 [5,46,47,48,49].

### 4.2. DM—A Major Risk Factor for CVD and Stroke

DM represents one of the most challenging health concerns, with CVD-related complications being the leading cause of death among affected patients. Therefore, it is essential to understand the relationship between DM and CVD and to adapt the pharmacological approach accordingly [45].

Studies estimate that, for patients suffering from T2DM, the risk of CVD is almost doubled; furthermore, the Northern Manhattan Study showed that the risk increases proportionally with the duration of DM, namely, each year of DM adds a 3% increase in the CVD risk [45]. The highest absolute CVD risk is observed in patients with target organ damage (TGO). T1DM also increases CVD risk; however, studies have identified several factors that further amplify this risk, namely, poor glycemic control, younger age at onset, and lower socioeconomic status [50].

The association between DM and stroke remains a major research focus. Evidence suggests that DM is a major risk factor for stroke (both ischemic and hemorrhagic), and almost one in four cases of stroke can be attributable to DM. However, when trying to determine whether DM influences recovery post-stroke, the conclusions are sometimes contradictory [51].

After analyzing the existent data about the effects of DM on stroke recurrence, a meta-analysis published in 2021 reached the conclusion that ischemic stroke recurrence is significantly higher for diabetic patients compared to those without DM [52].

It is difficult to estimate the percentage of strokes attributable to DM because DM is a complex condition that often coexists with other pathologies associated with a high CVD risk, such as hypertension, kidney dysfunction, and/or hypercholesterolemia. However, a meta-analysis concluded that the combined prevalence of DM among stroke patients is nearly 28%, which is higher than in the general population. Furthermore, the same article highlighted a higher prevalence of DM among patients with ischemic stroke, of approximately 33%, compared with those with hemorrhagic stroke (26%) [51].

### 4.3. Stroke—A Major Complication of DM

Stroke represents a major macrovascular complication of DM and a leading cause of morbidity and mortality. Chronic hyperglycemia, oxidative stress, inflammation, and endothelial damage are key pathological mechanisms linking the two conditions [44].

The two main dysfunctions that lay the foundation for DM are insulin resistance and β-cell dysfunction, both of which lead to abnormal blood glucose levels [53]. Pancreatic β-cell damage was initially fully attributed to cell death; however, more recent evidence shows that an excessive nutrient state, such as that observed in obese patients, leads to hyperglycemia and hyperlipidemia, which in turn induce oxidative stress. This oxidative state promotes chronic inflammation and ultimately damages cells within the islets of Langerhans [54]. These findings suggest that dysregulation of glucose levels, insulin secretion, and insulin action creates a self-perpetuating process driven by hyperglycemia [53].

Oxidative stress and, consequently, the chronic state of inflammation are triggered by hyperglycemia and hyperlipidemia and can also lead to endothelial dysfunction. Furthermore, endothelial dysfunction is a key feature that contributes to the development of micro- and macrovascular complications of DM, such as neuropathy, retinopathy, cardiomyopathy and atherosclerosis [55].

Accumulating evidence indicates that stroke in DM is a heterogeneous condition, with distinct pathophysiological substrates underlying different stroke subtypes [56,57,58]. In this regard, the contribution of DM to large artery atherosclerotic stroke appears to be primarily mediated by chronic hyperglycemia-driven processes, including advanced glycation end-product formation, oxidative stress, inflammation, dyslipidemia, and endothelial dysfunction, which collectively accelerate atherogenesis and promote plaque instability, thereby increasing the likelihood of large vessel occlusion [57,58]. By contrast, in cerebral small vessel disease and lacunar infarction, DM is more closely linked to chronic microangiopathic injury, characterized by endothelial dysfunction, BBB disruption, impaired vascular reactivity, and structural remodeling of small penetrating vessels, often in synergy with hypertension [59].

Cardioembolic stroke should be considered within a distinct mechanistic framework, as the influence of DM appears to be mediated predominantly through its association with cardiac structural and functional abnormalities, including atrial remodeling and diabetes-related cardiomyopathy, which may facilitate thrombus formation and embolization [56,57]. In contrast, hemorrhagic stroke reflects a different vascular milieu, more closely related to hypertension-driven arteriolar degeneration, increased vascular fragility, and alterations in hemostatic balance, rather than the atherosclerotic mechanisms that dominate in ischemic stroke [56].

These distinctions may have important implications for the interpretation of therapeutic effects. In particular, the observed or hypothesized benefits of GLP-1 RAs should not be assumed to be uniform across stroke subtypes. Their anti-atherosclerotic, anti-inflammatory, and endothelial-protective properties are likely to be most relevant in large artery disease, whereas potential neurovascular and microvascular effects may contribute in small vessel disease and selected ischemic contexts. Consistent with this, recent evidence suggests that GLP-1 RA therapy is associated with a reduction in stroke risk in patients with T2DM, although the relative contribution of specific mechanistic pathways remains to be fully elucidated [60,61].

Clinical evidence supports the following: (1) diabetic patients experiencing ischemic stroke exhibit more severe microvascular damage and worse outcomes [62]; (2) endothelial activation and inflammatory signaling are shared mechanisms underlying both renal and cerebral injury [62,63]; (3) BBB disruption and microvascular injury, reflected by parenchymal contrast enhancement and other imaging markers of BBB dysfunction, are amplified in the presence of hyperglycemia [64,65,66].

The clinical observations highlight the importance of early metabolic and anti-inflammatory intervention to prevent vascular complications in diabetic patients.

## 5. GLP1-RAs and GLP-1/GIP RAs as New Therapeutical Options for Stroke

### 5.1. GLP-1 RAs in Stroke Prevention

Evidence from large cardiovascular outcome trials (CVOTs) consistently indicates that GLP-1 RAs reduce major adverse cardiovascular events (MACEs), including stroke; however, the magnitude and consistency of this effect vary across agents and study populations. Importantly, stroke reduction is typically observed as part of a composite endpoint rather than as a primary outcome, which limits the ability to draw definitive cerebrovascular-specific conclusions [67,68].

Several landmark randomized controlled trials have evaluated the cardiovascular and cerebrovascular effects of GLP-1 RAs in patients with T2DM.

The SUSTAIN-6 trial, a randomized, double-blind, placebo-controlled cardiovascular outcomes study, evaluated the effects of semaglutide in patients with T2DM at high cardiovascular risk. Over a median follow-up of 2.1 years, semaglutide significantly reduced the incidence of MACEs compared to placebo. Notably, a substantial reduction in non-fatal stroke was observed, representing one of the most consistent cerebrovascular signals among GLP-1 RA trials. This finding is particularly relevant given that stroke reduction was not the primary endpoint, suggesting a potentially robust effect despite the trial not being specifically powered for this outcome. The mechanisms underlying this benefit are likely multifactorial, including improvements in glycemic control, weight reduction, blood pressure lowering, and anti-inflammatory and endothelial-protective effects. These results position semaglutide as one of the GLP-1 RAs with the strongest evidence for cerebrovascular risk reduction within the class [69].

However, this effect is not uniformly observed across all GLP-1 RAs. The REWIND trial, a large, randomized, double-blind, placebo-controlled cardiovascular outcomes study, evaluated the effects of dulaglutide in a relatively broad and representative population of patients with T2DM, the majority of whom did not have established CVD at baseline. Over a median follow-up of 5.4 years, dulaglutide was associated with a significant reduction in the composite endpoint of major adverse cardiovascular events compared to placebo. Importantly, this benefit included a statistically significant reduction in non-fatal stroke, making REWIND one of the few trials to demonstrate a clear cerebrovascular signal in a predominantly primary prevention population. This finding is particularly relevant, as it suggests that the protective effects of GLP-1 RAs may extend beyond high-risk individuals and may be applicable earlier in the course of the disease. The mechanisms underlying this benefit are likely multifactorial and include improvements in glycemic control, sustained weight reduction, modest blood pressure lowering, and favorable effects on endothelial function and systemic inflammation. Additionally, the long duration of follow-up in REWIND may have contributed to the detection of stroke reduction, as cerebrovascular benefits may require prolonged exposure to therapy. However, the magnitude of stroke risk reduction was relatively modest compared to that observed in SUSTAIN-6, and differences in study populations, baseline risk profiles, and pharmacokinetic properties of the agents may partially explain this variability [70].

Another major study, the LEADER trial, demonstrated that liraglutide reduced the risk of major cardiovascular events in patients with T2DM and high cardiovascular risk. Although the reduction in stroke as an individual endpoint did not reach statistical significance, this finding should be interpreted with caution. The trial was not specifically powered to detect differences in stroke outcomes alone, and the direction of effect was consistent with a potential benefit. From a clinical perspective, these results suggest that liraglutide should not be considered a stroke-specific therapeutic agent, but rather a treatment that reduces overall cardiovascular risk, within which stroke represents one component. Therefore, in patients specifically seeking cerebrovascular protection, liraglutide may still be appropriate as part of a broader cardiometabolic strategy, particularly when considering its beneficial effects on weight, glycemic control, blood pressure, and systemic inflammation. However, agents with more consistent stroke-specific signals, such as semaglutide or dulaglutide, may be preferred when cerebrovascular risk reduction is a primary therapeutic goal [71].

Comparative analysis of major trials suggests that, while stroke reduction may represent a class effect of GLP-1 RAs, important differences exist between individual agents. These differences may reflect variations in pharmacokinetic properties, receptor affinity, and patient selection across trials, highlighting that cerebrovascular benefit is likely influenced by both drug-specific and population-specific factors.

Meta-analyses of CVOTs further support the protective role of GLP-1 RAs against stroke. A large systematic review and meta-analysis including multiple randomized trials found that GLP-1 RAs significantly reduced the risk of total stroke and non-fatal stroke in patients with T2DM [60].

Another comprehensive meta-analysis confirmed that treatment with GLP-1 RAs was associated with a significant reduction in MACEs and stroke compared with placebo or standard care in patients with DM [67].

Similarly, an analysis published by Tsapas et al. demonstrated that GLP-1 RAs reduce the risk of stroke as part of their overall cardiovascular protective effect, supporting their use in patients with T2DM and high cardiovascular risk [68].

The consistent reduction in stroke risk observed in randomized trials and meta-analyses suggests that GLP-1 RAs may provide clinically meaningful cerebrovascular protection in patients with T2DM. These benefits appear to extend beyond glycemic control and likely involve improvements in weight, blood pressure, endothelial function, and systemic inflammation.

Consequently, current diabetes management guidelines recommend GLP-1 RAs for patients with T2DM who have established CVD or are at high cardiovascular risk, partly due to their demonstrated ability to reduce stroke and other cardiovascular events [67,68].

Recent reviews suggest that the stroke-protective effects of GLP-1 RAs can be understood within two complementary mechanistic domains: (i) prevention of ischemic events through systemic and vascular effects and (ii) modulation of ischemic injury through potential direct neuroprotective actions [72,73].

From a preventive perspective, GLP-1 RAs primarily act by modifying key vascular risk factors. These agents improve glycemic control, promote weight loss, lower blood pressure, and favorably influence lipid metabolism, thereby addressing several major drivers of atherosclerosis. In addition, they exert direct vasculoprotective effects, including improvement in endothelial function, attenuation of oxidative stress, and modulation of inflammatory pathways, which collectively contribute to plaque stabilization and reduced vascular injury. These systemic and vascular effects are considered the main mechanisms underlying the observed reduction in stroke risk in clinical settings [72,73].

In contrast, potential neuroprotective effects appear to involve direct actions within the central nervous system. Experimental evidence indicates that GLP-1 RAs may reduce infarct volume, neuronal apoptosis, excitotoxicity, and neuroinflammation, while preserving blood–brain barrier (BBB) integrity and promoting adaptive processes such as angiogenesis, neurogenesis, and neuroplasticity. However, these mechanisms have been primarily demonstrated in preclinical models, and their contribution to clinical stroke prevention or acute stroke outcomes in humans remains less clearly defined [72,73].

Taken together, current evidence supports a dual mechanistic framework in which GLP-1 RAs predominantly reduce stroke risk through systemic metabolic and vascular effects, while their direct neuroprotective actions represent a promising but not yet fully established pathway, the relevance of which may vary across clinical contexts. The distinct mechanisms underlying the effects of GLP-1 RAs in stroke prevention versus neuroprotection are summarized in Table 1 [72,73].

**Table 1:** Mechanisms of GLP-1 RAs in stroke.

Mechanistic Domain	Level	Key Mechanisms	Evidence
Metabolic and hemodynamic effects	Systemic	Improved glycemic control, weight reduction, ↓ blood pressure, improved lipid profile	Clinical
Vascular/anti-atherosclerotic effects	Vascular	Improved endothelial function, ↑ nitric oxide, ↓ oxidative stress and inflammation, plaque stabilization	Clinical + mechanistic
Renal effects	Systemic	Improved renal function	Clinical
Neuroprotection	CNS	↓ infarct volume, ↓ apoptosis, ↓ neuroinflammation	Preclinical
Neurovascular remodeling	CNS	BBB preservation, ↑ angiogenesis, ↑ neuroplasticity	Preclinical

*Abbreviations*: CNS—central nervous system; BBB—blood–brain barrier; ↓—decrease; ↑—increase.

Notably, several recent trials have extended the investigation of GLP-1 RAs to populations without overt diabetes, particularly individuals with overweight or obesity and established CVD. These findings suggest that the protective effects of GLP-1-based therapies on stroke risk are not exclusively mediated by glucose lowering. Instead, they likely reflect a broader impact on the metabolic–vascular axis, including weight reduction, improvement in endothelial function, attenuation of systemic inflammation, and modulation of atherosclerotic processes. Given that obesity is frequently associated with insulin resistance and subclinical metabolic dysfunction, these populations may still derive cerebrovascular benefit through mechanisms overlapping with those observed in patients with T2DM.

The available clinical evidence on GLP-1 RAs in stroke spans different stages of investigation, ranging from large completed CVOTs to early-phase and ongoing studies in acute stroke settings. It is important to distinguish between these levels of evidence to avoid overinterpretation. While robust data from completed trials and meta-analyses support the role of GLP-1 RAs in reducing overall cardiovascular risk, including stroke, evidence for their efficacy in acute stroke remains preliminary. Several ongoing trials, including those investigating semaglutide in the context of endovascular thrombectomy, have not yet reported results, and therefore no definitive conclusions can be drawn regarding their clinical benefit in this setting. These studies should be interpreted as hypothesis-generating and indicative of future research directions rather than established therapeutic strategies.

Taken together, the available evidence from CVOTs and meta-analyses supports a consistent role of GLP-1 RAs in reducing overall cardiovascular risk, including stroke, primarily in the context of primary and secondary prevention. However, the magnitude of stroke reduction varies across agents and is generally observed as part of composite endpoints rather than as a primary outcome. This suggests that the cerebrovascular benefits of these agents are likely mediated through systemic cardiometabolic improvements rather than direct, stroke-specific mechanisms.

### 5.2. GLP-1RAs in Acute Stroke: Emerging and Conflicting Evidence

While substantial evidence supports the role of GLP-1 RAs in long-term cardiovascular risk reduction and stroke prevention, their efficacy in the setting of acute ischemic stroke remains uncertain. This distinction is particularly important, as the mechanisms underlying chronic vascular protection differ from those involved in acute neuroprotection.

Table 2 summarizes the clinical trials using GLP-1 RAs and GLP-1/GIP RAs antidiabetic drugs already approved by the EMA and FDA and currently investigated for repurposing in stroke and stroke-related conditions.

**Table 2:** GLP-1 RAs and GLP-1/GIP RAs with potential cerebrovascular benefits—clinical trials summary.

Drug	Drug Class	Dosage and Route of Administration	Key Publications	ClinicalTrials ID Interventional/Observational (I/O)	DM as an Inclusion Criterion	Phase	Status
Semaglutide	GLP-1 RA	0.5 mg s.c. before or during EVT, and 7 days after the procedure; the patient will receive a total of 2 injections [74]	Wang et al. 2025 [75]	NCT05920889 [74] (I)	No	2	Completed
		0.5 mg s.c./week for 4 weeks [76]	No results posted	NCT05630586 [76] (I)	No	2	Recruiting
		0.24–2.4 mg s.c./week for 31–59 months [77]	Nicholls et al. 2026 [78] Deanfield et al. 2025 [79] Lubker et al. 2025 [80] McEwan et al. 2025 [81] Badve et al. 2025 [82] Scirica et al. 2024 [83] Deanfield et al. 2024 [84] Kosiborod et al. 2024 [85] Ryan et al. 2024 [86] Lincoff et al. 2023 [87] Leite et al. 2022 [88] Kanie et al. 2021 [89] Ryan et al. 2020 [90]	NCT03574597 [77] (I)	No	3	Completed
		0.5 mg s.c. during EVT [91]	No results posted	NCT07030621 [91] (I)	No	3	Not yet recruiting
		0.25–1 mg s.c./week for up to 52 weeks [92]	No results posted	NCT05780905 [92] (I)	Yes	4	Recruiting
		NA	No results posted	NCT06779929 [93] (O)	Yes	-	Active, not recruiting
		0.5 mg s.c. before and 1 week after EVT [94]	No results posted	NCT06788626 [94] (I)	No	3	Not yet recruiting
Liraglutide		0.6–1.8 mg/d s.c. for 90 days [95]	Zhu et al. 2026 [96]	NCT03948347 [95] (I)	Yes	-	Unknown status
Dulaglutide		NA	No results posted	NCT07282041 [97] (I)	No	2, 3	Recruiting
			No results posted	NCT06779929 [93] O)	Yes	-	Active, not recruiting
Exenatide		5 μg s.c., single dose [98]	No results posted	NCT02829502 [98] (I)	No	2	Unknown status
		5 μg s.c./twice daily for five days, commencing within 9 h of symptom onset [99]	Bladin et al. 2023 [100]	NCT03287076 [99] (I)	No	2	Unknown status
Tirzepatide	GLP-1/GIP RA	NA	No results posted	NCT06779929 [93] (O)	Yes	-	Active, not recruiting

*Abbreviations*: I—interventional, O—observational; GLP-1 RA—glucagon-like peptide-1 (GLP-1) receptor agonist; mg/d—milligrams daily, EVT—endovascular thrombectomy.

*Semaglutide*

The phase 2, multicenter, randomized, pilot study identified by the ID NCT05920889 [74] on Clinical Trials.gov aimed to investigate the effects of semaglutide in patients with stroke caused by large vessel occlusion. The enrollment for this study was 140 patients of both sexes who were treated with reperfusion therapy, with an LKW-to-puncture time of less than 12 h, aged 18–100 years. The results of the study suggest that semaglutide is safe for use in patients with large vessel occlusion; moreover, patients (that were not receiving intravenous thrombolysis) treated with semaglutide showed improved neurological outcomes [75].

An ongoing multicenter, randomized trial (ClinicalTrials.gov identifier: NCT05630586 [76]) is aiming to evaluate the safety and efficacy of semaglutide in non-diabetic patients who suffered from acute stroke with disabling neurological deficits and an LKW of less than 4.5 h. It is estimated that the study will have 380 participants aged ≥18 years, both males and females.

The trial registered on ClinicalTrials.gov with the ID NCT03574597 [77] explored the cardiovascular outcomes of semaglutide when administered to patients who already had established CVD and obesity (with a body mass index (BMI) ≥ 27 kg/m^2^). The study was conducted on 17,604 patients of all sexes with ages of 45 years or older, and the results were published in several articles. According to Nicholls et al. [78], semaglutide was linked to a significant reduction in the total number of hospitalizations; moreover, the patients treated with semaglutide that were hospitalized spent fewer days in the hospital. Deanfield et al. [84] highlighted that the group treated with semaglutide showed improved outcome measures in patients with heart failure with both preserved and reduced ejection fraction (HFpEF and HFrEF) compared to patients without heart failure. The findings of Deanfield et al. [79] include the fact that the administration of semaglutide was associated with significantly lower risks of MACEs; the cardioprotective properties of semaglutide were not dependent on the baseline adiposity. Scirica et al. [83] reported that among the group treated with semaglutide during the trial there were lower rates of all-cause death. Lincoff et al. [87] concluded that semaglutide lowered the risk of non-fatal stroke and non-fatal myocardial infarction as well as the risk of death from cardiovascular causes in patients with pre-existing CVD who were overweight or obese. Lübker et al., Badve et al., and Leite et al. reported a significant reduction in cardiovascular event risk among patients treated with semaglutide in the trial [80,82,88]. Kosiborod et al. found that, in patients with HFpEF, semaglutide was associated with a significant reduction in the risk of the composite endpoint of cardiovascular death or heart failure events, as well as worsening heart failure events, but did not significantly reduce cardiovascular mortality alone [85]. Ryan et al. showed that semaglutide treatment can reduce MACEs in patients with overweight or obesity and established CVD without DM [86,90]. McEwan et al. assessed the cost-effectiveness of semaglutide in the SELECT trial and reported that, per 100,000 subjects, treatment prevented 2791 non-fatal myocardial infarctions, 3000 coronary revascularizations, 487 non-fatal strokes, and 115 cardiovascular deaths [81]. Kanie et al., in a meta-analysis including multiple clinical trials, among which was the SELECT trial, reported that GLP-1 RAs were associated with a reduction in cardiovascular and all-cause mortality, as well as stroke, while showing no significant effect on myocardial infarction or heart failure hospitalization [89].

A randomized, double-blind trial identified by the ID NCT07030621 [91] on ClinicalTrials.gov aims to evaluate the efficacy of semaglutide regarding the improvement of neurological outcomes in patients with acute stroke after endovascular thrombectomy. The study is expected to enroll 436 participants of all sexes with ages ≥ 18 years old.

An ongoing clinical study (ClinicalTrials.gov identifier: NCT05780905 [92]) will explore the effects of semaglutide on intracranial blood flow and on BBB permeability for an estimated number of 50 patients with T2DM, of all sexes, with ages between 40 and 65 years old.

An ongoing study registered on ClinicalTrials.gov with the ID NCT06779929 [93] is aiming to compare the effects of tirzepatide, dulaglutide and semaglutide on MACEs in diabetic patients. The estimated enrollment is 70,000 participants of both sexes with ages ≥ 40 years old.

An ongoing study (ClinicalTrials.gov identifier: NCT06788626 [94]) will look into the effects of semaglutide in patients with large vessel occlusion stroke who received endovascular treatment. The study will include an estimated 390 individuals of all sexes aged 18 or older. Participants should have a National Institute of Health Stroke Scale (NHISS) ≥ 6 at the time of brain imaging.

*Liraglutide*

The clinical trial registered on ClinicalTrials.gov with the ID NCT03948347 [95] aimed to explore the effect of liraglutide on individuals with acute minor ischemic stroke (AMIS) or high-risk transient ischemic attack (TIA). The enrollment of this study was 1708 participants of all sexes with ages of 50 years or older. Zhu et al. [96] concluded that liraglutide might lower the risk of stroke recurrence and improve stroke outcomes among Chinese patients with either AMIS or TIA.

*Dulaglutide*

The influence of dulaglutide on cerebral hemodynamics will be analyzed in patients with mild stroke but severe stenosis or TIA and impaired cardiovascular risk within the previous three months of acute stroke or TIA. The estimated enrollment for this study is 130 patients of all sexes with ages between 21 and 80 years old [97]. Another clinical study [93] is aiming to compare the effects of tirzepatide, semaglutide and dulaglutide on MACEs for patients with T2DM. The estimated enrollment for the study is 70,000 patients of all sexes with ages ≥ 40 years old.

*Exenatide*

The clinical trial identified by the ID NCT02829502 [98] will assess the effect of exenatide on cerebral blood flow velocity in patients with ischemic stroke (radiologically confirmed stroke); the estimated number of participants is 30, the patients will be aged 18 or older, and all sexes are eligible. The modified ranking scale (mRS) should be less than 2 prior to onset of symptoms and the NHISS should be between 1 and 20 at the onset of symptoms.

A trial registered in 2021 with the ID NCT03287076 [99] aimed to compare exenatide versus standard care in acute ischemic stroke; the study included 350 participants, males and females, 18 years or older. Participants were asked to have a CT that excluded hemorrhagic stroke, and blood glucose levels on admission were ≥4 mmol/L. The first trial treatment was administered within 9 h of stroke onset. The conclusion of the trial was that exenatide did not exert any beneficial effect on neurological impairment at 7 days in individuals with ischemic stroke [100].

*Tirzepatide*

Tirzepatide is currently being evaluated in an ongoing clinical trial (ID: NCT06779929) in comparison with dulaglutide and semaglutide to assess their effects on MACEs in patients with DM. No results have been published to date [93].

A key inconsistency in the current clinical evidence is the discrepancy between the neutral results observed with exenatide in the TEXAIS trial and the more promising signals reported in early-phase studies with semaglutide. Several factors may explain these divergent findings. First, there are important pharmacokinetic differences between these drugs. Exenatide works for a short period of time, with a relatively limited duration of action. In contrast, semaglutide has a long-lasting effect and keeps the receptors activated for a longer time, which may be more relevant for effects on the nervous system and blood vessels. Second, differences in patient selection may have contributed to outcome variability. The TEXAIS trial included a broad population of patients with acute ischemic stroke, whereas ongoing semaglutide studies are often focused on more selected populations, such as patients with large vessel occlusion undergoing endovascular thrombectomy, in whom reperfusion-related mechanisms may interact with drug effects. Third, timing of administration is likely critical. In TEXAIS, exenatide was administered within a wider therapeutic window (up to 9 h), potentially after irreversible ischemic injury had occurred, whereas semaglutide is being investigated in closer temporal association with reperfusion therapies, where neuroprotective strategies may have greater impact. Taken together, these differences suggest that the efficacy of GLP-1 RAs in acute stroke may depend on drug-specific properties, patient stratification, and the precise timing of intervention, rather than representing a uniform class effect.

In contrast to the robust evidence regarding prevention, data on acute ischemic stroke remain limited and, in some cases, contradictory. While exenatide failed to demonstrate a significant improvement in neurological outcomes, early-phase studies with semaglutide suggest potential benefits in selected patient populations. These discrepancies highlight the importance of factors such as pharmacokinetic profile, patient selection, and timing of administration, suggesting that the effects of GLP-1 RAs in acute stroke are not uniform and may depend on specific clinical contexts.

### 5.3. Emerging Therapies and Ongoing Trials

Newer potential therapeutical agents that are in line for approval and for which current data available on ClinicalTrials.gov indicate a greater possibility of success for treating CVDs (including stroke) are included in Table 3.

**Table 3:** Antidiabetic agents with potential cardiovascular benefits currently being evaluated in clinical studies.

Agent	Mechanism of Action	Dosage and Route of Administration	ClinicalTrials ID Interventional/Observational (I/O)	DM as an Inclusion Criterion	Phase	Status	Studied Conditions
CagriSema	Amylin–GLP-1 RA combination	2.4 mg/2.4 mg s.c. (once weekly) [101]	NCT05669755 [101] (I)	Yes	3	Active, not recruiting	CVD
Maridebart cafraglutide	GLP-1 RA—GIP receptor antagonistic antibody	NA s.c	NCT07037433 [102] (I)	No	3	Recruiting	ASCVD, obesity, overweight
Orforglipron	Small-molecule, oral GLP-1 RA	NA orally	NCT07223593 [103] (I) *	No	3	Recruiting	PAD
			NCT06948422 [104] (I) *	No	3	Recruiting	HT
			NCT05803421 [105] (I) *	Yes	3	Active, not recruiting	CVD, CKD
Retatrutide	GLP-1, GIP and glucagon triple receptor agonist	NA s.c.	NCT05882045 [106] (I)	No	3	Active, not yet recruiting	Obesity, CVD

* Patients who had a stroke within 60–90 days prior to screening were excluded from the trial. *Abbreviations*: I—interventional, O—observational, CagriSema—combination of cagrilintide and semaglutide, PAD—peripheral arterial disease, CVD—cardiovascular disease, ASCVD—Atherosclerotic Cardiovascular Disease, HT—hypertension, CKD—Chronic Kidney Disease, NA—not available.

*CagriSema*

The safety and efficacy of CagriSema (cagrilintide in combination with semaglutide) will be evaluated in patients with established CVDs (including ischemic or hemorrhagic stroke). The study will enroll 7101 participants, both males and females, with ages ≥ 55 or older and a BMI over 25 kg/m^2^. Individuals with T2DM are also eligible for this trial if their HbAc is between 6.5 and 10% [101].

*Maridebart cafraglutide*

The cardiovascular effects of Maridebart cafraglutide will be analyzed in individuals with a history of ASCVD (including prior stroke). The estimated enrollment is 12,800 patients, of all sexes, with ages between 45 and 99 years old and a BMI ≥ 27 kg/m^2^ [102].

*Orforglipron*

Orforglipron, an oral, non-peptide GLP-1 RA, was recently FDA-approved for obesity [107] and is under investigation for T2DM and cardiovascular indications [108].

The efficacy and safety of orforglipron will be evaluated for patients with PAD (with intermittent claudication of Fountaine Stage II) and who have an ankle brachial index (ABI) ≤ 0.9. The estimated enrollment for this study is 1205; participants of all sexes are eligible for the study if they are 18 or older [103]. Also, orforglipron’s safety and efficacy will be explored in patients with hypertension, untreated or on stable medication for more than 30 days before enrollment. Participants should have a BMI over 25 kg/m^2^. The study will have 974 participants of all sexes with ages ≥ 18 years [104].

Another trial aims to compare orforglipron with insulin glargine in obese or overweight patients (with a BMI ≥ 25 kg/m^2^ and a stable weight for at least 90 days prior to screening) over 18 years old with T2DM and increased cardiovascular risk. In order to be eligible for this study, patients must be under treatment with at least one but no more than three antihyperglycemic drugs. The actual enrollment of the study reaches 2749 patients [105].

*Retatrutide*

Retatrutide’s efficacy and safety will be investigated in patients with severe obesity (BMI ≥ 35 kg/m^2^) and CVDs (including prior ischemic or hemorrhagic stroke). Participants must report at least one unsuccessful dietary effort to reduce their weight. The study has an estimated enrollment of 1800 participants of all sexes with ages ≥ 18 years old [106].

An important distinction that emerges from the current literature is the difference between stroke prevention and acute stroke management. Most of the robust clinical evidence supporting GLP-1 RAs relates to primary and secondary prevention, where long-term improvements in glycemic control, body weight, blood pressure, and lipid profile contribute to reduced cerebrovascular risk [72,73]. In contrast, evidence supporting their role in acute ischemic stroke remains limited and inconclusive. For example, clinical studies evaluating exenatide in acute stroke did not show significant improvement in short-term neurological outcomes [100], and ongoing trials with semaglutide are still in their early phases or have not yet reported definitive results [74,75,76,91,92,93,94]. This highlights a critical gap between mechanistic plausibility and clinical efficacy in the acute setting.

Another relevant aspect is the comparison of different therapies. While semaglutide, liraglutide, and dulaglutide have demonstrated cardiovascular benefits in large-scale trials, differences in molecular structure, receptor affinity, pharmacokinetics, and potency may translate into variable cerebrovascular effects. Semaglutide appears to have a more pronounced effect on weight reduction and possibly stroke risk [69], whereas liraglutide shows broader cardiovascular benefits without a clearly dominant effect on stroke alone [71]. Dulaglutide has demonstrated efficacy in a population with a lower baseline cardiovascular risk [70], suggesting potential differences in patient selection and therapeutic impact. Tirzepatide has shown superior metabolic effects, particularly in weight reduction [19], but the direct impact on stroke outcomes remains insufficiently characterized. Therefore, it remains unclear whether dual or triple agonists will provide additive cerebrovascular protection beyond established GLP-1 RAs.

In addition, the current literature is heavily focused on ischemic stroke, with limited data on hemorrhagic stroke. Several factors may explain this gap. Hemorrhagic stroke is less prevalent than ischemic stroke and is associated with higher acute mortality, which makes patient recruitment and trial design more challenging. Furthermore, concerns regarding safety and bleeding risk often lead to the exclusion of patients with prior or acute hemorrhagic stroke from randomized controlled trials, contributing to the scarcity of evidence. From a mechanistic perspective, the effects of GLP-1 RAs on hemorrhagic stroke risk remain incompletely understood. DM is characterized by a prothrombotic state, endothelial dysfunction, and vascular remodeling, which may contribute to both ischemic and hemorrhagic cerebrovascular events. GLP-1 RAs have been shown to improve endothelial function, reduce oxidative stress, and attenuate inflammatory pathways, which could theoretically stabilize the vascular wall and reduce fragility. In addition, although these agents are not primarily anticoagulant or antiplatelet drugs, indirect effects on platelet function and coagulation pathways have been suggested, potentially contributing to a more balanced hemostatic profile. Importantly, current clinical data do not indicate an increased risk of hemorrhagic stroke associated with GLP-1 RAs, although dedicated analyses are lacking. Some ongoing trials include patients with a history of hemorrhagic stroke, which may provide further insight into their safety profile in this population. From a clinical perspective, in the absence of robust evidence, GLP-1 RAs should be considered primarily for their overall cardiometabolic benefits, while their specific role in patients with prior hemorrhagic stroke remains to be clarified, highlighting the need for dedicated studies in this high-risk population.

Given the distinct underlying mechanisms, including vascular fragility and bleeding risk, it cannot be assumed that the benefits of incretin-based therapies extend equally to hemorrhagic stroke.

Finally, several key gaps in knowledge remain. First, there is a lack of dedicated randomized trials specifically designed to evaluate stroke as a primary endpoint in patients treated with incretin-based therapies. Second, the role of these agents in acute stroke and early post-stroke recovery is still unclear. Third, the comparative effectiveness of different GLP-1 RAs and dual or triple agonists has not been adequately addressed. Fourth, the long-term impact of these therapies on neurological function, cognitive outcomes, and stroke recurrence requires further investigation.

Although incretin-based therapies represent a promising strategy for reducing cerebrovascular risk in patients with DM, current evidence is predominantly driven by indirect cardiovascular benefits observed in prevention settings. Their role in acute stroke and their potential for direct neuroprotection remain to be clearly established, highlighting the need for targeted, mechanism-driven clinical trials.

A substantial proportion of the available data on acute stroke are derived from ongoing or early-phase trials, further emphasizing the need for cautious interpretation.

Overall, current evidence indicates that GLP-1 RAs should be primarily regarded as agents for long-term vascular risk reduction rather than as established therapies for acute stroke. While emerging data raise the possibility of direct neuroprotective effects, these remain to be confirmed in adequately powered randomized trials. Future research should focus on identifying patient subgroups most likely to benefit and on clarifying whether specific agents offer advantages in acute cerebrovascular settings.

## 6. Conclusions

The relationship between DM and stroke is complex, multifactorial, and clinically significant, with DM acting as both a major risk factor for stroke occurrence and a determinant of poorer post-stroke outcomes. Advances in antidiabetic pharmacotherapy, particularly the development of incretin-based therapies, have introduced new perspectives in the prevention of cerebrovascular disease. Current evidence from large CVOTs support the role of GLP-1 RAs in reducing MACEs, including stroke, primarily in the context of primary and secondary prevention.

The available data indicate that the reduction in stroke risk associated with GLP-1-based therapies is largely mediated by indirect mechanisms, including improvements in glycemic control, body weight, blood pressure, and systemic inflammation.

Emerging therapies, including dual and triple incretin receptor agonists, such as tirzepatide and retatrutide, as well as novel agents like orforglipron and combination strategies (e.g., CagriSema), offer promising metabolic and cardiovascular benefits. Nevertheless, their specific impact on cerebrovascular outcomes, particularly stroke incidence and recovery, has not been adequately established.

While incretin-based therapies represent a promising approach for reducing cerebrovascular risk in patients with DM, their role remains primarily preventive rather than therapeutic in the context of acute stroke. Future research should focus on well-designed randomized controlled trials with stroke-specific endpoints, direct comparisons between agents, and the evaluation of neurological and functional outcomes. A better understanding of the mechanisms underlying potential neuroprotection will be essential to determine whether these therapies can be effectively integrated into stroke management strategies.

## Data Availability

No new data were created in this study.
